# The impact of cytomegalovirus infection on new-onset diabetes mellitus after kidney transplantation: a review on current findings

**DOI:** 10.12860/jnp.2014.27

**Published:** 2014-10-01

**Authors:** Behzad Einollahi, Mohsen Motalebi, Mahmood Salesi, Mehrdad Ebrahimi, Mehrdad Taghipour

**Affiliations:** ^1^Nephrology and Urology Research Center, Baqiyatallah University of Medical Sciences, Tehran, Iran

**Keywords:** Cytomegalovirus, New-Onset diabetes, Kidney transplantation

## Abstract

*Context:* New onset diabetes mellitus after transplantation (NODAT) increases the risk of cardiovascular disease, rate of infections, graft rejection and graft loss as well as decreases patient and graft survival rates. There is a controversy surrounding the impact of cytomegalovirus (CMV) infection in the development of NODAT. This meta-analysis aims to identify the role of CMV infection leading to the development of NODAT in kidney recipient patients.

*Evidence Acquisitions:* We searched several electronic databases, including PubMed, Embase, Medline, Scopus, Trip Database and Google Scholar for studies that completely fulfill our criteria between January 1990 and January 2014

*Results:* Seven studies with 1389 kidney transplant patients were included in this metaanalysis.The mean age of patients ranged from 42.8 to 48.8 years and males made up 53% to 75% of patients in the cohort studies. The incidence of NODAT varies from 14.3% to 27.1% in these studies. Overall adj OR was 1.94 [exp (0.66)] with a 95% CI of 1.26-2.98 [exp (0.23) and (1.09)]. There was no significant publication bias based on the Begg’s and Egger’s test (p value = 0.17 and 0.54, respectively).

*Conclusions:* Our study showed that CMV infection is a risk factor for increasing incidence of NODAT. Thus, prophylaxis against CMV infection after kidney transplantation is strongly suggested. However, further clinical trials and cohorts are needed to confirm this association.

**Implication for health policy/practice/research/medical education:**

New onset diabetes mellitus after transplantation (NODAT) is a complicated disorder which can lead to serious complications such as graft rejection in renal transplant recipients. Understanding the etiology of NODAT can help in prevention.

## 1. Context

New onset diabetes mellitus after transplantation (NODAT) is a well-known complication following solid organ transplantation and has been defined as the start of diabetes mellitus after transplantation in non-diabetic individuals. It has been reported that the incidence of NODAT ranges from 2% to 53% in renal transplant recipients (1). It has been shown that NODAT increases the risk of cardiovascular disease (2), rate of infections (3), graft rejection, graft loss (2) as well as decreased patient and graft survival rates (4).

Many risk factors have been found to have an influence on the development of NODAT. In 1985 Lehr *et al* (5) reported a case of cytomegalovirus (CMV) induced NODAT in a kidney recipient patient, after that the role of CMV infection in NODAT has been an area of interest to researchers. Since then other studies have supported (6,7) the relationship between them whilst other studies (8,9) have failed to prove this association. However, the influence of CMV infection on developing NODAT has still remained a question. If the impact of CMV infection on higher incidence of NODAT is proven, initiating prophylaxis against CMV infection after transplantation will be strongly suggested (10). Thus, our aim was to conduct a meta-analysis to answer the question about the role of CMV infection on developing NODAT in kidney recipient patients based on available literature.

## 2. Evidence acquisition

We searched electronic databases, including PubMed, Embase, Medline, Scopus, Trip database and Google Scholar, for studies between January 1990 and January 2014 to identify those that reported the effect of CMV infection on occurrence of diabetes mellitus among kidney transplant recipients. Our keywords searched in all field of articles included “new-onset diabetes”, “post-transplant diabetes”, “diabetes mellitus”, “cytomegalovirus”, “kidney transplant” and their abbreviations as well as synonyms. We also searched Current contents (institute for scientific information) and Cochrane library with a focus on clinical trials registry to reduce publication bias. In order to identify further relevant articles, references of pertinent articles, the identified papers and qualitative topic reviews were also reviewed. All of the search methods were done separately by two investigators and the results were compared to eliminate duplicate reports. All final eligible studies, based on following inclusion criteria, were qualitatively assessed by STROBE statement for cohort studies (11) and CONSORT 2010 checklist for randomized trial (12) and none of them were identified as low quality studies. The STROBE statement evaluates 22 items in manuscripts, each item has one score and total score is 22. A score less than 12 identified as low quality. We restricted our search to human studies and placed no limitations on language.

### 2.1. Inclusion criteria

To be included in this meta-analysis, a study had to fulfill following criteria: 1) be a clinical trial or cohort [retrospective or prospective]; 2) enrolled patients more than 18 years of age with no prior history of diabetes mellitus; 3) followed patients for at least three months after renal transplantation with <10% loss to follow up; as well as 4) provided data on CMV infections in patients with and without NODAT. We included all studies with these criteria’s regardless of their results.

### 2.2. Ineligible studies

Studies that enrolled patients with combined organ transplants, individuals on maintenance hemodialysis and where there were reports of inadequate data were not considered for in our analysis. In addition, the studies including all individuals using prophylaxis against CMV infection were excluded. Case reports, letters and review articles were also excluded.

### 2.3. Data extraction

We extracted the following information from each study: study’s characteristics (study and first author name, year of publication, study location, type of study and number of participants) and participants’ characteristics (age, sex, BMI, family history of DM, dialysis duration and deceased or live donor). We selected the adjusted OR (adj OR) and 95% confidence intervals (CI) for NODAT after kidney transplantation in CMV infection and non CMV infection patients as our primary outcome measure in this analysis. The adjOR had been reported in five (6,7,9,13,14) of seven (6,7,9,13-16) studies; however, one of these articles (13) had not described 95% CI. We also calculated by univariate analysis the un-adj OR (and 95% CI) for NODAT after RTx in CMV infection and non CMV infection patients; this was the secondary outcome measure in our meta-analysis.

### 2.4. Definitions

NODAT was diagnosed according following criteria: fasting blood glucose (FBS) levels higher than 126 mg/dL on two separate occasions; random blood sugar >200 mg/dL, confirmed by FBS >126 mg/dL, and 2-hour post-prandial blood sugar >200 mg/dL, confirmed by FBS >126 mg/dL or 2-hour plasma glucose ≥200 during an oral glucose tolerance test [OGTT, using a glucose load containing the equivalent of 75-g anhydrous glucose dissolved in water (17)]. Alternatively, DM was defined as the requirement of glucose lowering medications (insulin or oral hypoglycemic agents for >3 month).

### 2.5. Data analysis

We extracted existing adj OR and 95% CI in four of seven articles and used logarithm of adj ORs (Log OR) and their 95% CI for less bias and converted to OR again after obtaining pooled Log OR for primary output. Data for NODAT after RTx in patients with and without CMV infection in all seven studies were also used to calculate un-adj OR (and 95% CI) as secondary output. A chi-squared test was used for heterogeneity and a p value ≤0.1 represented significant heterogeneity. The random mantel-haenszel model was used in significant heterogeneity; otherwise, we applied the fixed mantel-haenszel model to achieve pooled OR. In case of heterogeneity, we examined meta-regression for age, BMI and sex separately to see whether sub group analysis is beneficial. We did not enter other variables in meta-regression because of small sample size of studies and not reporting of other variables in several articles included in our analysis. Funnel plot figure was examined visually and also Begg’s rank correlation test and Egger’s regression asymmetry test were used to identify publication bias (p value ≤0.05 was considered as significant publication bias). All analysis procedures were done by STATA statistical software version 11 for windows.

## 3. Results

### 3.1. Literature search

All of search results after deleting duplicate records were 4831 studies. A total of 4736 irrelevant studies were excluded by primary title evaluation. Abstract review led to exclude 35 other unrelated studies. Subsequently, 29 irrelevant papers were removed after a thorough review of the full paper. Twenty-four articles out of 31 related studies could not meet the inclusion criteria that are as following: eighteen articles were excluded for type of articles [review articles: 14 (1,18-30), case report: two (5,31), commentary: one (32) and cross-sectional: one (33)], 4 studies did not provide data on CMV infections in patients with and without NODAT (8,34-36), one study enrolled pediatrics patients (37) and one study had used the prophylaxis regimens against CMV in all patients (38). Finally, 7 studies with 1389 kidney transplant patients, which completely fulfill the inclusion criteria, were included in this meta-analysis.

### 3.2. Patient characteristics

Some important demographic and clinical characteristics of the studies are shown in Tables 1-3. Two studies were from Asia and five were from Europe (Table 1). The mean age of patients ranged from 42.8 to 48.8 years and males made up 53 to 75 percent of patients in the cohorts (Tables 1 and 2). There was a higher proportion of CMV infection and NODAT in Valderhaug *et al*., and Marin *et al*., studies, respectively (Table 3).

**Table 1:** Baseline characteristics of studies included in the meta-analysis

**Reference number**	**Study design**	**Country**	**Patients number**	**Age (years)**	**Male (%)**
6	Co, P	Norway	173	48.0±16	69
7	Co, P	Norway	124	48.0±15	75
9	Co, R	Romania	177	42.8±12.2	66
13	Co, R	Taiwan	43	44.2±10.4	51
14	Co, P	Norway	494	48.8±15.1	65
15	Co, R	Poland	308	47.3±12.7	60
16	Co, R	Japan	70	45.0±11.1	53

Co: cohort study; P: prospective; R: retrospective

**Table 2:** Baseline characteristics of studies included in the meta-analysis

**Reference number**	**BMI** **at RTx**	**Family history for DM (%)**	**Duration of dialysis (months)**	**Deceased donor (%)**	**Immunosuppressive regimen**
6	23.5±3.8	20.0	NR	NR	CS, CsA, AZA
7	23.2	21.0	NR	NR	CS, CsA, AZA
9	23.0±4.18	13.0	28.8±34	97.2	CS, CsA (or TAC), AZA (or MMF)
13	NR	7.0	NR	60.1	CS, CsA (or TAC), AZA (or MMF)
14	24.0±3.67	NR	NR	60.0	CS, CsA (or TAC), MMF
15	23.8±3.78	26.2	24.8±27.95	100.0	CS, CsA (or TAC), AZA (or MMF)
16	23.0±3.57	NR	59.4±60.58	NR	CS, TAC, AZA (or MMF)

RTx: renal transplantation; BMI: body mass index; DM: diabetes mellitus; NR: not reported; CS: corticosteroids; CsA: cyclosporine; AZA: azathioprine; TAC: tacrolimus; MMF: mycophenolate mofetil.

**Table 3:** Baseline characteristics of studies included in the meta-analysis

**Reference number**	**CMV infection (%)**	**NODAT** **(%)**	**Diagnostic criteria for** **NODAT**
6	45 (26)	31 (17.2)	FBS≥7.7 mmol/l or 2-h pp≥11.1 mmol/l
7	61 (49.2)	20 (16.1)	According to an OGTT (39)
9	57 (32.2)	48 (27.1)	According to ADA (40)
13	5 (11.6)	9 (21)	According to ADA (40)
14	281 (56.9)	77 (15.6)	FBS≥7 mmol/l or 2-h glucose ≥11.1 mmol/l during an OGTT (41)
15	47 (15.2)	72 (23.4)	According to ADA (42)
16	10 (14.3)	10 (14.3)	HbA1c continuously ≥6.5 mg/dl, FBS≥126 or requiring hypoglycemic agent ≥3 months

CMV, cytomegalovirus; NODAT, new-onset diabetes mellitus, FBS; fasting blood sugar, OGTT; oral glucose tolerance test

### 3.3. Primary and secondary outcomes

**Figure 1:**
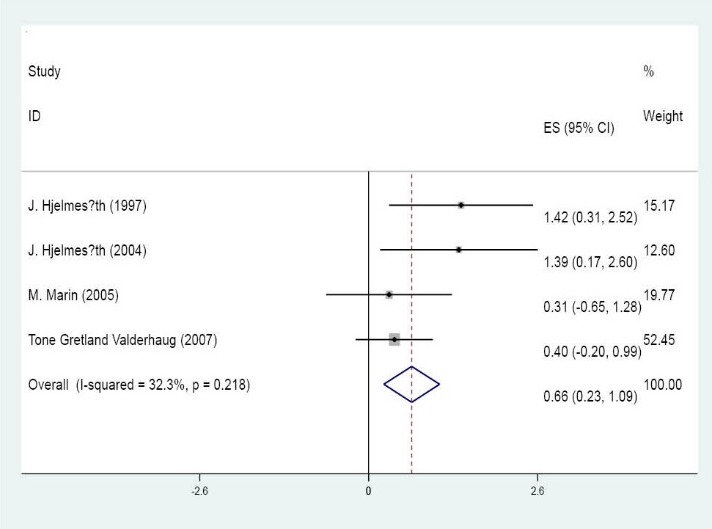
Forest plot of CMV impact on developing NODAT using logarithm of adjusted ORs (ES) and their 95% CI

Table 4 shows the results of primary outcome. Chi-squared test (Q test) detected no significant differences in homogeneity (p=0.2, I_2_=32.3%). In a fixed model, overall adj OR was 1.94 [exp (0.66)] with a 95% CI of 1.26-2.98 [exp (0.23) and (1.09)] (Figure 1). There was not a significant publication bias according to Begg’s and Egger’s test (p= 0.17 and 0.54, respectively). We also depicted funnel plot figure and found that it was relatively symmetric. Overall estimated un-adj OR was 2.11 with a 95% CI of 1.28-3.49 (Table 5) in a random model (p value of homogeneity=0.06, I_2_=49.5% and Tau_2_=0.19). There was no significant difference for males, mean age and BMI in meta-regression model (p=0.2, 0.9 and 0.6, respectively). Significant publication bias was not detected in begg’s test (p=0.4) and egger’s test (p=0.3).

**Table 4:** CMV infection and NODAT: Log OR and 95% CI

**Reference number**	**Log OR**	**95% CI**	**Weight (%)**
6	1.42	0.31 – 2.52	15.17
7	1.39	0.17 – 2.60	12.60
9	0.31	-0.65 – 1.28	19.77
14	0.40	-0.20 – 0.99	52.45
Overall fixed effect model	0.66	0.23 – 1.09	100.00

**Table 5:** CMV infection and NODAT: un-adj OR and 95% CI

**Reference number**	**OR**	**95% CI**	**Weight (%)**
6	2.48	1.10 - 5.60	17.51
7	5.24	1.64 - 16.77	11.82
9	2.35	1.17 - 4.73	20.02
13	8.00	1.09 - 58.54	05.29
14	2.11	1.24 - 3.59	23.92
15	0.86	0.41 - 1.84	18.75
16	0.22	0.01 - 4.22	02.70
Overall random effect model	2.11	1.28 - 3.49	100.00

## 4. Discussion

Many viruses such as entroviruses, rubella, mumps, Epstein-Barr virus, varicella zoster and hepatitis C virus have been shown to have effect on type 1 and 2 DM (43). Although, CMV is a risk factor for type 1 DM (44), its impact on NODAT has remained elusive. In this meta-analysis, we found that the risk of NODAT in kidney transplants with CMV infection was 1.94 fold more as compared to individuals without CMV infection using adj ORs from the studies. This significant relationship was proved by overall pooled OR using un-adj ORs. There was a difference in the result of evaluated studies in term of CMV induced NODAT. Though, three studies (15,16) reported no significant relationship between CMV infection and NODAT; other studies (6,7,13) detected CMV infection as a risk factor for NODAT. In addition, Valderhaug and coworkers (14) only found the relationship between CMV infection and NODAT in univariate analysis whilst a multivariate analysis, adjusted for age, prednisolone, type of cohort, HLA-B27 phenotype and BMI did not support this association. We believe that the following points explain this difference in the results of the studies included in our meta-analysis:

Firstly, the studies used different criteria to identify CMV infection. Isolation of the CMV virus and detection of viral proteins or nucleic acid are different ways to recognize CMV infection. In addition, active systemic CMV infection can be diagnosed as CMV-DNA in plasma by polymerase chain reaction methods or by the detection of CMV-antigenemia in leukocytes (i.e., CMVpp65) (22). Four from seven works in our analysis did not report criterion for identifying CMV infection (6,13,15,16). Three remaining studies used different criterion to recognize CMV infection; Hjelmesaeth *et al* (7) defined CMV infection as one or more CMV pp65 antigen-positive cells per 100,000 leucocytes, Marin and colleagues (9) defined it as more than 50 infected cells per 200,000 leukocytes using the pp65 assay or isolation of CMV antigenemia or fourfold increase in the baseline IgG and Valderhaug *et al* (14) diagnosed it by CMV-pp65 antigen in leukocytes or CMV-DNA in plasma, but they did not report details. Thus using various criteria and methods with different sensitivity and specificity can lead to an overestimate or may in fact underestimate CMV infection in the studies. Thus, more studies using more accurate diagnostic methods such as CMV-PCR are required for assessing the CMV infection as a risk factor for NODAT.

Secondly, altered criteria for diagnosis of NODAT were defined in the studies (Table 4). There were no unique criteria to identify NODAT and determine its’ actual incidence in various works. Only three (9,13,15) of seven studies used unique criteria for identification of NODAT according to ADA. Therefore different NODAT criteria may be one of the reasons for various incidence of NODAT in the studies.

Furthermore, the impact of immunosuppression regimens especially steroids, tacrolimus and cyclosporine on developing NODAT have been shown in previous studies. (6,45,46). The role of glococorticoids on developing NODAT was due to the stimulation of glucogenesis and the impairment of glucose uptake by addipose tissue and muscle which would lead to insulin resistance. Furthermore, some investigators have shown that glococorticoids can supress insulin secretion and stimulate islet cell apoptosis at higher doses (47,48). Hjelmesaeth *et al.* have shown that daily prednisolone dose, the presence of hypertension, the number of antihypertensive agents used and the use of diuretics or b-blockers, all were associated with insulin resistance 3 months after transplantation (49). In addition, it has been shown that steroid tapering or withdrawal improved insulin resistance (50-52) and on the other hand pulse steroid therapy for acute rejection was the most important risk factor for NODAT (53).

There is a controversy on the role of cyclosporine on NODAT in the literature. A recent experimental study (54) reported that cyclosporine can impair insulin sensitivity. Although, an increase in the incidence of NODAT has been shown during cyclosporine era (55,56), other studies (6,57,58) showed that the incidence of NODAT decreased with cyclosporine. This can be due to reduction of corticosteroid dosage after that cyclosporine was started. On the other hand, cyclosporine can reduce corticosteroid liver metabolism and increase its blood level. However, it has been shown cyclosporine alone can increase developing NODAT (59,60). Several studies (61,62) reported higher incidence of NODAT in individuals using tacrolimus rather than cyclosporine. Tacrolimus binds to the FK506 binding protein and inhibits the calcineurin in beta cells and suppresses insulin secretion at insulin mRNA transcription (63). Radu *et al* also showed the inhibitory effect of tacrolimus on insulin secretion was the result of reduced ATP production and glycolysis due to decreased glucokinase activity (64). However, tacrolimus impaired glucose metabolism in most studies (13,64,65), Valderhaug and coworkers (14) showed the lower incidence of NODAT in group with higher dosage of tacrolimus compared to other group using significant lower dosage of that. In conclusion applying different immunosuppressive regimens can be another factor for different outcomes in studies.

Finally, various risk factors except CMV infection for NODAT have been described in the literature such as older age, black ethnicity and Hispanic patients rather than white patients (19), overweight or obese individuals (66), hepatitis C virus (67), family history of type 2 diabetes (6), pre-transplant FBS level (68), some genetic variants (69), ethnicity (66), hypomagnesaemia (70), male donor (19), immunosuppressant agents (66), polycystic kidney disease (71) and others. Each study has evaluated some of these risk factors and they evaluated the association of CMV and NODAT adjusting for different variables. Thus, there may be some confounders that affect the results of each study.

Several mechanisms have been suggested to explain the impact of CMV on diminishing insulin secretion as following: β cell damaging directly by CMV infection and apoptosis or by infiltrative leukocyte or by induction of pro-inflammatory cytokine. Also, some studies (49) showed the impact of CMV infection in increase of insulin resistance. However; Hjelmesaeth et al (7) found that this virus did not have any influence on insulin resistance. It seems this difference may be due to different steroid protocol and tapering in studies. The steroids impact on insulin resistance has been showed in several investigations (72,73). A Norwegian study (74) showed that prednisolone below 7 mg/day led to improve insulin action. However, it seems, further studies need to understand the actual influence of CMV infection on insulin resistance.

There were some limitations in our meta-analysis. There was a lack of homogeneity in the seven studies included in the analysis in terms of CMV diagnostic method, immunosuppression regimens and dosage and method of their tapering, diagnostic criteria for NODAT and dissimilation of factors adjusted in the studies. In addition, it was not clear in the studies weather patients first caught CMV infection and then NODAT or vice versa. However, there were not many studies on the effect of CMV infection linked to NODAT and we had to select our inclusion criteria less stringently, so these limitations were unavoidable.

## 5. Conclusions

Despite all the limitations in the studies, our study, based on currently available data showed that CMV infection is a risk factor for increasing the incidence of NODAT. However, further homogenous studies in future are required to confirm this finding. Meanwhile, based on the results of our meta-analysis we strongly support the use of CMV prophylaxis in renal transplant recipients to diminish the incidence of NODAT.

## Conflict of interests

The authors declare no conflict of interest.

## Authors’ contributions

Writing paper: MM, BE and MT. Searching the literature: MM and MB. Analysis: MS and MM. Supervision: EB.

## Acknowledgments

The authors thank Dr. Abdol-Karim Mobasher for preparing full paper of needed articles.

## Funding/Support

None.

## References

[1] Kesiraju S, Paritala P, Rao Ch UM, Sahariah S (2014). New onset of diabetes after transplantation - An overview of epidemiology, mechanism of development and diagnosis. Transpl Immunol.

[2] Hjelmesaeth J, Hartmann A, Leivestad T, Holdaas H, Sagedal S, Olstad M (2006). The impact of early-diagnosed new-onset post-transplantation diabetes mellitus on survival and major cardiac events. Kidney Int.

[3] Pietrzak-Nowacka M, Safranow K, Dziewanowski K, Debska-Slizien A, Glyda M, Golembiewska E (2008). Impact of posttransplant diabetes mellitus on graft function in autosomal dominant polycystic kidney disease patients after kidney transplantation. Ann Acad Med Stetin.

[4] Valderhaug TG, Hjelmesaeth J, Jenssen T, Roislien J, Leivestad T, Hartmann A (2012). Early posttransplantation hyperglycemia in kidney transplant recipients is associated with overall long-term graft losses. Transplantation.

[5] Lehr H, Jao S, Waltzer W, Anaise D, Rapaport F, editors editors (1985). Cytomegalovirus-induced diabetes mellitus in a renal allograft recipient. Transplant Proc.

[6] Hjelmesaeth J, Hartmann A, Kofstad J, Stenstrom J, Leivestad T, Egeland T (1997). Glucose intolerance after renal transplantation depends upon prednisolone dose and recipient age. Transplantation.

[7] Hjelmesaeth J, Sagedal S, Hartmann A, Rollag H, Egeland T, Hagen M (2004). Asymptomatic cytomegalovirus infection is associated with increased risk of new-onset diabetes mellitus and impaired insulin release after renal transplantation. Diabetologia.

[8] Sulanc E, Lane JT, Puumala SE, Groggel GC, Wrenshall LE, Stevens RB (2005). New-onset diabetes after kidney transplantation: an application of 2003 International Guidelines. Transplantation.

[9] Marin M, Renoult E, Bondor C, Kessler M, editors editors (2005). Factors influencing the onset of diabetes mellitus after kidney transplantation: a single French center experience. Transplant Proc.

[10] Nemati E, Taheri S, Pourfarziani V, Einollahi B (2008). Cytomegalovirus disease in renal transplant recipients: an Iranian experience. Exp Clin Transplant.

[11] von Elm E, Altman DG, Egger M, Pocock SJ, Gotzsche PC, Vandenbroucke JP (2007). The Strengthening the Reporting of Observational Studies in Epidemiology (STROBE) statement: guidelines for reporting observational studies. PLoS Med.

[12] Schulz KF, Altman DG, Moher D (2010). CONSORT 2010 statement: updated guidelines for reporting parallel group randomized trials. Ann Intern Med.

[13] Yang W-C, Chen Y-S, Hsieh W-C, Shih M-H, Lee M-C (2006). Post-transplant Diabetes Mellitus in Renal Transplant Recipients—Experience in Buddhist Tzu Chi General Hospital. Tzu Chi Med J.

[14] Valderhaug TG, Hjelmesaeth J, Rollag H, Leivestad T, Roislien J, Jenssen T (2007). Reduced incidence of new-onset posttransplantation diabetes mellitus during the last decade. Transplantation.

[15] Madziarska K, Weyde W, Krajewska M, Patrzalek D, Janczak D, Kusztal M (2011). The increased risk of post-transplant diabetes mellitus in peritoneal dialysis-treated kidney allograft recipients. Nephrol Dial Transplant.

[16] Numakura K, Satoh S, Tsuchiya N, Horikawa Y, Inoue T, Kakinuma H (2005). Clinical and genetic risk factors for posttransplant diabetes mellitus in adult renal transplant recipients treated with tacrolimus. Transplantation.

[17] Alberti K, Davidson MB, DeFronzo RA, Drash A, Genuth S, Harris MI (1998). Report of the expert committee on the diagnosis and classification of diabetes mellitus. Diabetes care.

[18] Bodziak KA, Hricik DE (2009). New-onset diabetes mellitus after solid organ transplantation. Transpl Int.

[19] Ghisdal L, Van Laecke S, Abramowicz MJ, Vanholder R, Abramowicz D (2012). New-onset diabetes after renal transplantation: Risk assessment and management. Diabetes care.

[20] Guitard J, Rostaing L, Kamar N (2011). New-onset diabetes and nephropathy after renal transplantation. Contrib Nephrol.

[21] Hjelmesaeth J, Asberg A, Muller F, Hartmann A, Jenssen T (2005). New-onset posttransplantation diabetes mellitus: insulin resistance or insulinopenia? Impact of immunosuppressive drugs, cytomegalovirus and hepatitis C virus infection. Curr Diabetes Rev.

[22] Hjelmesaeth J, Muller F, Jenssen T, Rollag H, Sagedal S, Hartmann A (2005). Is there a link between cytomegalovirus infection and new-onset posttransplantation diabetes mellitus? Potential mechanisms of virus induced beta-cell damage. Nephrol Dial Transplant.

[23] José Pérez-Sola M, José Castón J, Solana R, Rivero A, Torre-Cisneros J (2008). Indirect effects of cytomegalovirus infection in solid organ transplant recipients. Enferm Infecc Microbiol Clin.

[24] Pham PT, Pham PM, Pham SV, Pham PA, Pham PC (2011). New onset diabetes after transplantation (NODAT): an overview. Diabetes Metab Syndr Obes.

[25] Pham PTT, Pham PCT, Lipshutz GS, Wilkinson AH (2007). New Onset Diabetes Mellitus After Solid Organ Transplantation. Endocrinol Metab Clin North Am.

[26] Rodrigo E, Fernandez-Fresnedo G, Valero R, Ruiz JC, Pinera C, Palomar R (2006). New-onset diabetes after kidney transplantation: risk factors. J Am Soc Nephrol.

[27] Sagedal S, Hartmann A, Rollag H (2005). The impact of early cytomegalovirus infection and disease in renal transplant recipients. Clin Microbiol Infect.

[28] Türk TR, Witzke O (2011). New-onset diabetes mellitus after kidney transplantation. Der Nephrologe.

[29] Markell M (2004). New-onset diabetes mellitus in transplant patients: pathogenesis, complications, and management. Am J Kidney Dis.

[30] Davidson JA, Wilkinson A (2004). New-Onset Diabetes After Transplantation 2003 International Consensus Guidelines An endocrinologist’s view. Diabetes care.

[31] Leung Ki EL, Venetz JP, Meylan P, Lamoth F, Ruiz J, Pascual M (2008). Cytomegalovirus infection and new-onset post-transplant diabetes mellitus. Clin Transplant.

[32] Smith RM (2004). CMV prophylaxis: a useful step towards prevention of post-transplant diabetes?. Diabetologia.

[33] Chan HW, Cheung CY, Liu YL, Chan YH, Wong HS, Chak WL (2008). Prevalence of abnormal glucose metabolism in Chinese renal transplant recipients: A single centre study. Nephrol Dial Transplant.

[34] Gourishankar S, Jhangri GS, Tonelli M, Wales LH, Cockfield SM (2004). Development of diabetes mellitus following kidney transplantation: a Canadian experience. Am J Transplant.

[35] Sharma RK, Prakash R, Jeloka T, Gupta A, Gulati S, Sharma AP (2003). Posttransplant diabetes mellitus in renal transplant recipients-a single-center experience. Transplant Proc.

[36] Woodward RS, Schnitzler MA, Baty J, Lowell JA, Lopez-Rocafort L, Haider S (2003). Incidence and Cost of New Onset Diabetes Mellitus Among US Wait-Listed and Transplanted Renal Allograft Recipients. Am J Transplant.

[37] Burroughs TE, Swindle JP, Salvalaggio PR, Lentine KL, Takemoto SK, Bunnapradist S (2009). Increasing incidence of new-onset diabetes after transplant among pediatric renal transplant patients. Transplantation.

[38] Cole EH, Prasad GV, Cardella CJ, Kim JS, Tinckam KJ, Cattran DC (2013). A pilot study of reduced dose cyclosporine and corticosteroids to reduce new onset diabetes mellitus and acute rejection in kidney transplant recipients. Transplant Res.

[39] (2000). Report of the Expert Committee on the Diagnosis and Classification of Diabetes Mellitus. Diabetes Care.

[40] (2003). Report of the expert committee on the diagnosis and classification of diabetes mellitus. Diabetes Care.

[41] Consultation WHO. Definition, diagnosis and classification of diabetes mellitus and its complications: Part 1; diagnostic and classification of Diabetes Mellitus 1999. 10.1002/(SICI)1096-9136(199807)15:7<539::AID-DIA668>3.0.CO;2-S 9686693

[42] (2004). Diagnosis and Classification of Diabetes Mellitus. Diabetes care.

[43] Fabrizi F, Lampertico P, Lunghi G, Mangano S, Aucella F, Martin P (2005). Review article: hepatitis C virus infection and type-2 diabetes mellitus in renal diseases and transplantation. Aliment Pharmacol Ther.

[44] Pak C, Mcarthur R, Eun H-M, Yoon J-W (1988). Association of cytomegalovirus infection with autoimmune type 1 diabetes. Lancet.

[45] Kamar N, Mariat C, Delahousse M, Dantal J, Al Najjar A, Cassuto E (2007). Diabetes mellitus after kidney transplantation: a French multicentre observational study. Nephrol Dial Transplant.

[46] Vincenti F, Friman S, Scheuermann E, Rostaing L, Jenssen T, Campistol J (2007). Results of an international, randomized trial comparing glucose metabolism disorders and outcome with cyclosporine versus tacrolimus. Am J Transplant.

[47] Huscher D, Thiele K, Gromnica-Ihle E, Hein G, Demary W, Dreher R (2009). Dose-related patterns of glucocorticoid-induced side effects. Ann Rheum Dis.

[48] Koizumi M, Yada T (2008). Sub-chronic stimulation of glucocorticoid receptor impairs and mineralocorticoid receptor protects cytosolic Ca2+ responses to glucose in pancreatic beta-cells. J Endocrinol.

[49] Hjelmesaeth J, Midtvedt K, Jenssen T, Hartmann A (2001). Insulin resistance after renal transplantation: impact of immunosuppressive and antihypertensive therapy. Diabetes Care.

[50] Hagen M, Hjelmesaeth J, Jenssen T, Morkrid L, Hartmann A (2003). A 6-year prospective study on new onset diabetes mellitus, insulin release and insulin sensitivity in renal transplant recipients. Nephrol Dial Transplant.

[51] Lemieux I, Houde I, Pascot A, Lachance JG, Noel R, Radeau T (2002). Effects of prednisone withdrawal on the new metabolic triad in cyclosporine-treated kidney transplant patients. Kidney Int.

[52] Boots JM, van Duijnhoven EM, Christiaans MH, Wolffenbuttel BH, van Hooff JP (2002). Glucose metabolism in renal transplant recipients on tacrolimus: the effect of steroid withdrawal and tacrolimus trough level reduction. J Am Soc Nephrol.

[53] Pascual J, Zamora J, Galeano C, Royuela A, Quereda C (2009). Steroid avoidance or withdrawal for kidney transplant recipients. Cochrane Database Syst Rev.

[54] Lopes P, Fuhrmann A, Sereno J, Pereira MJ, Nunes P, Pedro J (2013). Effects of cyclosporine and sirolimus on insulin-stimulated glucose transport and glucose tolerance in a rat model. Transplant Proc.

[55] Menegazzo LA, Ursich MJ, Fukui RT, Rocha DM, Silva ME, Ianhez LE (1998). Mechanism of the diabetogenic action of cyclosporin A. Horm Metab Res.

[56] Dmitrewski J, Krentz AJ, Mayer AD, Buckels JA, Barnes AD, Smith J (2001). Metabolic and hormonal effects of tacrolimus (FK506) or cyclosporin immunosuppression following renal transplantation. Diabetes Obes Metab.

[57] Sumrani NB, Delaney V, Ding ZK, Davis R, Daskalakis P, Friedman EA (1991). Diabetes mellitus after renal transplantation in the cyclosporine era--an analysis of risk factors. Transplantation.

[58] von Kiparski A, Frei D, Uhlschmid G, Largiader F, Binswanger U (1990). Post-transplant diabetes mellitus in renal allograft recipients: a matched-pair control study. Nephrol Dial Transplant.

[59] Roth D, Milgrom M, Esquenazi V, Fuller L, Burke G, Miller J (1989). Posttransplant hyperglycemiaIncreased incidence in cyclosporine-treated renal allograft recipients. Transplantation.

[60] Yoshimura N, Nakai I, Ohmori Y, Aikawa I, Fukuda M, Yasumura T (1988). Effect of cyclosporine on the endocrine and exocrine pancreas in kidney transplant recipients. Am J Kidney Dis.

[61] Cole EH, Johnston O, Rose CL, Gill JS (2008). Impact of acute rejection and new-onset diabetes on long-term transplant graft and patient survival. Clin J Am Soc Nephrol.

[62] Shah T, Kasravi A, Huang E, Hayashi R, Young B, Cho YW (2006). Risk factors for development of new-onset diabetes mellitus after kidney transplantation. Transplantation.

[63] Tamura K, Fujimura T, Tsutsumi T, Nakamura K, Ogawa T, Atumaru C (1995). Transcriptional inhibition of insulin by FK506 and possible involvement of FK506 binding protein-12 in pancreatic beta-cell. Transplantation.

[64] Radu RG, Fujimoto S, Mukai E, Takehiro M, Shimono D, Nabe K (2005). Tacrolimus suppresses glucose-induced insulin release from pancreatic islets by reducing glucokinase activity. Am J Physiol Endocrinol Metab.

[65] Boots JM, van Duijnhoven EM, Christiaans MH, Wolffenbuttel BH, van Hooff JP (2002). Glucose metabolism in renal transplant recipients on tacrolimus: the effect of steroid withdrawal and tacrolimus trough level reduction. J Am Soc Nephrol.

[66] Kesiraju S, Paritala P, Rao Ch UM, Sahariah S (2014). New onset of diabetes after transplantation - an overview of epidemiology, mechanism of development and diagnosis. Transpl Immunol.

[67] Fabrizi F, Martin P, Dixit V, Bunnapradist S, Kanwal F, Dulai G (2005). Post-transplant diabetes mellitus and HCV seropositive status after renal transplantation: meta-analysis of clinical studies. Am J Transplant.

[68] Cosio FG, Kudva Y, van der Velde M, Larson TS, Textor SC, Griffin MD (2005). New onset hyperglycemia and diabetes are associated with increased cardiovascular risk after kidney transplantation. Kidney Int.

[69] Voight BF, Scott LJ, Steinthorsdottir V, Morris AP, Dina C, Welch RP (2010). Twelve type 2 diabetes susceptibility loci identified through large-scale association analysis. Nat Genet.

[70] Talmud PJ, Hingorani AD, Cooper JA, Marmot MG, Brunner EJ, Kumari M (2010). Utility of genetic and non-genetic risk factors in prediction of type 2 diabetes: Whitehall II prospective cohort study. BMJ.

[71] Hamer RA, Chow CL, Ong AC, McKane WS (2007). Polycystic kidney disease is a risk factor for new-onset diabetes after transplantation. Transplantation.

[72] Andrews RC, Walker BR (1999). Glucocorticoids and insulin resistance: old hormones, new targets. Clin Sci (Lond).

[73] Geer EB, Islam J, Buettner C (2014). Mechanisms of Glucocorticoid-Induced Insulin Resistance: Focus on Adipose Tissue Function and Lipid Metabolism. Endocrinol Metab Clin North Am.

[74] Midtvedt K, Hjelmesæth J, Hartmann A, Lund K, Paulsen D, Egeland T (2004). Insulin resistance after renal transplantation: the effect of steroid dose reduction and withdrawal. J Am Soc Nephrol.

